# The Effects of Hyperbaric Oxygen at Different Pressures on Oxidative Stress and Antioxidant Status in Rats

**DOI:** 10.3390/medicina55050205

**Published:** 2019-05-24

**Authors:** Şefika Körpınar, Hafize Uzun

**Affiliations:** 1 Department of Underwater and Hyperbaric Medicine, Faculty of Medicine, Çanakkale Onsekiz Mart University, Çanakkale 17020, Turkey; 2 Department of Biochemistry, Cerrahpasa Medical Faculty, Istanbul University Cerrahpasa, Istanbul 34098, Turkey; huzun59@hotmail.com

**Keywords:** hyperbaric oxygen, malondialdehyde, superoxide dismutase, glutathione

## Abstract

*Background*: The optimal use of oxygen at greater than atmospheric pressures in any operational or therapeutic application (hyperbaric oxygen, HBO_2_) requires awareness of the fact that the beneficial effects of oxygen coexist with toxic effects depending on the pressure and duration of exposure. In this study, we aimed to investigate the effect of HBO_2_ therapy on oxidative stress and antioxidant status in commonly used protocol for acute HBO_2_ indications, such as carbon monoxide intoxication, central retinal artery occlusion, crush injury, gas gangrene, and to compare it with normobaric oxygen (NBO_2_) in healthy rats. *Materials and Methods*: Fifty-six male, young adult Wistar albino rats were randomly divided into seven groups and named as Group I through Group VII. Plasma malondialdehyde (MDA), superoxide dismutase (SOD), and erythrocyte glutathione (GSH) levels in control group were compared to the levels in other groups. *Results*: The increases in MDA levels and the decrease in SOD activities were statistically significant in HBO_2_ groups at the end of the first 24 h when compared to the control group, and the significant decrease in erythrocyte GSH level was only at 2.4 atmospheres absolute. *Conclusions*: The present study showed that pressure and frequency of exposure are important factors to consider when investigating HBO_2_-induced oxidative stress and antioxidant response.

## 1. Introduction

Oxygen therapy (in atmospheric conditions, 1 atmosphere absolute (ATA), 760 mmHg, normobaric oxygen, NBO_2_) is one of the first and basic applications in many acute treatment algorithms. The justification for its inclusion in these algorithms derives from the request to prevent tissues from the hypoxia. Albeit the targeted therapy the same as NBO_2_, hyperbaric oxygen (HBO_2_) treatment is limited to indications determined by international organizations, such as Undersea and Hyperbaric Medical Society and European Committee for Hyperbaric Medicine, compared with the wide use of NBO_2_ [1,2,3]. The toxic effect of oxygen was foreseen by Joseph Priestly during its discovery. However, it took almost two centuries until Gershman and colleagues elucidated the molecular mechanism of this toxicity in rats exposed to HBO_2_ [4,5]. The practical demands imposed by the development of military underwater operations using closed-circuit oxygen rebreathers to carry out reconnaissance, blasting, and other tasks or submarine wreck escape training provided further impetus for this research [6]. Today, it is well accepted that exposure to HBO_2_, namely breathing 100% oxygen at greater than atmospheric pressure (1 ATA), increases the formation of reactive oxygen species (ROS), which in turn results in consumption of antioxidants and reduces antioxidant enzyme activity, eventually causing lipid peroxidation, organ injury, and DNA damage [7,8,9,10,11,12,13,14,15,16,17]. 

The deleterious effects of HBO_2_ may occur on the central nervous system (Paul Bert effect), on the lung (Lorrain Smith effect), and on the genome [16,18,19]. Since the early 1940s, it is also well known that prolonged exposure to NBO_2_ has caused ocular toxic effects [20]. ROS, however, also trigger essential physiological processes that would not be considered stressful to the cell or organism and adaptive responses. Previous studies showed that NBO_2_ and HBO_2_ treatment may also serve as an in vivo model for the investigation of the redox signaling [17,21,22]. The different effects of NBO_2_ and HBO_2_ on the cell signal transmission cascades or pathways for a variety of growth factors, cytokines, and hormones point out the various paradox, refractory period, and different response times [17,23]. The study designs were usually established as a single session of acute HBO_2_ exposure in high pressures and durations. The most commonly used treatment protocol in clinical practice, however, is daily exposures to 2.0–2.4 ATA for 90–120 min for 20–40 days as in the diabetic foot infections or chronic refractory osteomyelitis [17]. In these prolonged repetitive exposures, although clinical reports are controversial due to commonly conducted on patients with various pathologies in which oxidative stress has already existed [10,24], experimental studies in healthy rats without any clinical pathology suggest that a cumulative oxidative effect [25]. On the other hand, much more intensive (three times in the first 24 h and once or twice daily thereafter) and short-term (24 h to 10 days) treatment protocols are preferred in clinical conditions that are life-threatening or causing the loss of tissue and function, such as carbon monoxide intoxication, central retinal artery occlusion, crush injury, compartment syndrome, gas gangrene, and necrotizing soft tissue infections [26,27,28,29]. In such acute indications, the most important reason why HBO_2_ should not be administered at much more frequent intervals and in longer sessions is the potential risk of oxygen toxicity. In this study, we aimed to evaluate oxidative stress and antioxidant status at the end of the first 24 h and the tenth day, in such intensive therapeutic protocols, and to compare it with NBO_2_ in healthy rats.

## 2. Materials and Methods

### 2.1. Animals

The Experimentation Ethics Committee of Cerrahpasa Faculty of Medicine approved the experimental procedures of the study and the study is registered to National Thesis Center with the number of 157001 on 13 August 2004. Fifty-six male young adult Wistar albino rats weighing 180–260 g were randomly divided into seven groups and named Group I through Group VII. The animals were housed in 35 x 50 cm cages, in a room with the temperature regulated at 21 ± 2 °C, humidity 45%–50%, and light/dark cycles of 12 h. Food and water were provided ad libitum. All animals were fed a commercial diet during the course of the experiment.

### 2.2. HBO_2_ and NBO_2_ Administration 

HBO_2_ and NBO_2_ exposures were conducted in a small research chamber (0.4 m^3^) at the Department of Underwater and Hyperbaric Medicine, Istanbul Faculty of Medicine. The chamber was flushed with oxygen for 10 min to vent the air inside before HBO_2_ and NBO_2_ administration so that the animals could breathe 100% oxygen. The HBO_2_ sessions were 60 min at 2.0 and 2.4 ATA, excluding 10 min of compression and 10 min of decompression. NBO_2_ was given for 60 min inside the chamber, without pressurization. The procedures for each group were as follows.

Group-IControl group, no oxygen was given.Group-IIThree sessions of NBO_2_ were administered at six-hour intervals within 24 h.Group-IIIThree sessions of HBO_2_ at 2 ATA were administered at six-hour intervals within 24 h.Group-IVThree sessions of HBO_2_ at 2.4 ATA were administered at six-hour intervals within 24 h.Group-VTotal of 15 sessions of NBO_2_ were administered in 10 days as; 3 times a day on the first day (6-h intervals), two times a day on the second, third and fourth day (10-h intervals), and once a day for the last six days (22-h intervals).Group-VITotal of 15 sessions of HBO_2_ at 2 ATA were administered in 10 days as scheduled for Group V.Group-VIITotal of 15 sessions HBO_2_ were administered as scheduled for Group V, but at 2.4 ATA.

### 2.3. Preparation of Plasma and Erythrocyte Lysates

Right after the exposure period, the rats were anesthetized with a ketamine–xylazine combination and their chests were opened by median sternotomy. Heparinized blood samples were obtained by cardiac puncture and immediately transported in a cooler with ice to the laboratory. Upon arrival at the laboratory, plasma samples were separated by centrifugation (4 °C, 2500 g, 10 min) and divided into 0.5–1.0 mL aliquots, placed in cryovials and stored at –80 °C until analyzed. Erythrocytes were washed three times with cold phosphate buffer saline (PBS) (0.9% NaCl, 10 mM Na_2_HPO_4_, and pH = 7.4) after removal of plasma, buffy coat and upper 15% of cells, and glutathione (GSH) was studied in the same day.

### 2.4. Biochemical Assays

#### 2.4.1. Measurement of Malondialdehyde (MDA) Levels

Lipid peroxidation status was ascertained by formation of MDA as an end product of fatty acid peroxidation. MDA levels were measured in plasma using the methodology of Buege and Aust [30]. This method was used to obtain a spectrophotometric measurement of the color produced during the reaction to thiobarbituric acid with MDA at 535 nm.

#### 2.4.2. Measurement of Cu–Zn-Superoxide Dismutase (Cu–Zn-SOD)

Plasma Cu–Zn-SOD activity was determined using the method of Sun et al. [31]. The assay involves inhibition of nitroblue tetrazolium (NBT) (Sigma Chemical Co., St. Louis, U.S.) and reduction with xanthine/xanthine oxidase (Sigma Chemical Co.), which is used as a superoxide generator. One unit of SOD is defined as the amount of protein that inhibits the rate of NBT reduction by 50%.

#### 2.4.3. Measurement of Erythrocyte Glutathione (GSH) Levels

Reduced GSH concentration was determined according to the method of Beutler et al. [32] using metaphosphoric acid for protein precipitation and 5-5′-dithiobis-2-nitrobenzoic acid for color development. The GSH concentration was calculated with a molar absorption coefficient ε = 1.36 × 10^−4^ M^−1^ cm^−1^ at a wavelength λ = 412 nm. The concentration of total haemoglobin was determined in hemolysate using a conventional method with Drabkin’s reagent, and a standard curve is read at 546 nm in a spectrophotometer [33].

### 2.5. Statistical Analyses

Plasma MDA, SOD, and erythrocyte GSH levels in control group were compared to the levels in other groups. The levels between the groups that received oxygen at particular pressure for 24 h and for 10 days were also compared (i.e., the levels between II and V, III and VI, IV and VII). Data were assessed for normality using Shapiro–Wilk test and Q–Q plot, boxplot, and histogram graphics. Statistical evaluations were done by Kruskal–Wallis one-way analysis of variance (ANOVA) analysis. The Dunnett test was used for comparison of the control group with the other six groups, and the Wilcoxon test (significant with *p* < 0.05) was used for comparing parameters in the groups that received HBO_2_ or NBO_2_ for 24 h and 10 days.

## 3. Results

The levels of plasma MDA and SOD activity, and erythrocyte GSH levels are shown in Table 1. The increases in MDA levels and the decrease in SOD activities were statistically significant in Groups III and IV when compared to the control group. The erythrocyte GSH levels decreased significantly only in Group IV compared to the control group. At the end of the tenth day, no significant difference was found in any of the groups compared to the control group.

The comparison of the groups in each protocol that received NBO_2_ or HBO_2_ for 24 h and 10 days are shown in Table 2. The decreases in MDA levels in Groups V, VI and VII (which received NBO_2_ or HBO_2_ for 10 days) were statistically significant when compared to the levels in Groups II, III and IV (which received NBO_2_ or HBO_2_ for 24 h), respectively. SOD activity in Group V (NBO_2_ for 10 days) significantly decreased when compared to Group II (NBO_2_ for 24 days). In the other two HBO_2_ protocols, however, SOD activities were increased significantly when the levels detected after 24 h (Groups III and IV) were compared with the levels detected after 10 days (Groups VI–VII). The increase in GSH levels was significant only in Group VII when compared to Group IV.

## 4. Discussion

The main findings of this study were that (i) three sessions of HBO_2_ exposures in the first 24 h cause significant oxidative stress with an increase in MDA levels, a decrease in SOD activity, and a decrease in erythrocyte GSH levels only in 2.4 ATA, (ii) despite continued exposures on subsequent days these effects were resolved at the end of the tenth day and, (iii) there were no such effects observed with NBO_2_. 

Oxygen, like any other drug available, can be both lifesaving and detrimental, depending on how it is used. At pressures greater than 3 ATA, the primary concern of oxygen inhalation is neurotoxicity that is known as the “Paul Bert effect”. Therefore, the measures to prevent this effect are mainly limiting oxygen pressure and exposure time [18,34]. In the present study, 2.0 and 2.4 ATA were used because of the usually preferred pressure values for monoplace and multiplace chamber in clinical practice. The results indicate that three sessions of HBO_2_ administration created significant oxidative stress in twenty-four hours at both pressure values. Previous studies showed that single HBO_2_ exposure caused oxidative DNA base modifications in healthy human volunteers’ leukocytes, however, this oxidative DNA damage was detected only after the first HBO_2_ session, not after further treatments under the same conditions [8,14]. On the other hand, the DNA of lymphocytes taken from divers exposed to long-term repetitive HBO_2_ exhibited increased oxidative susceptibility when exposed to an additional single session of HBO_2_ in vitro [7]. In healthy rat lungs, a cumulative oxidative effect has been reported after the fifteenth session accompanied by an increase in antioxidant enzyme activity [25]. James Lorain Smith, a pathologist, noticed this effect as “pneumonia from oxygen” on the fourth day in rats that were exposed to 73% oxygen at 1 ATA, while trying to produce the “Paul Bert effect” [18,19]. In 1970, Clark and Lambertsen suggested that decreases in vital capacity could be used to predict the onset, rate of development, and degree of severity of these toxic process in the lung caused by oxygen exposure [35]. Subsequently, Bardin and Lambertsen developed an equivalent dose concept: The Unit Pulmonary Toxicity Dose (UPTD) [36]. However, as in the case of the study designs, one of the most important limitations of the UPTD approach is also related to the calculations, as they do not take into consideration “recovery” periods between exposures to oxygen at toxic levels. The significance of chronic, long-term effects of repeated exposure to subclinical or mild degrees of pulmonary intoxication, represent another serious limitation [37]. Namely, the degrees of oxygen tolerance for a single pattern of intermittent HBO_2_ exposure varies among different enzyme systems, and even the same enzyme systems in different organs (lung, brain, and leukocyte) [38]. Therefore, in this study, we preferred venous blood samples to make a systematic evaluation.

At the end of the 10-day exposure period, MDA levels seem to return to baseline values, hence, significant decreases were observed in both NBO_2_ and HBO_2_ exposure when the MDA levels at the end of the first 24 h and tenth day were compared. These decreases in MDA levels were accompanied by the increase in SOD activity. At GSH levels, there was a statistically significant increase only in 2.4 ATA exposure (Table 2). Several studies have shown that HBO_2_ exposure induces antioxidant activity [11,13,17,21,25]. Global gene expression analysis of human endothelial cells after HBO_2_ exposure at 2.4 ATA—the same pressure as in our study Groups IV and VII—revealed upregulation of antioxidant, cytoprotective, and immediate-early genes through a number of cellular pathways, including Nrf2, integrin, and ERK/MAPK. This increase corresponds to increased resistance to a lethal oxidative stress. These cells are a direct target of HBO_2_ during wound healing. Analysis of gene array data also showed that HBO_2_ stimulates endothelial cell proliferation, an important component of angiogenesis and wound healing, through the activation of growth-regulatory genes. The ability of HBO_2_ to enhance endogenous antioxidant enzymes that suppress ROS-induced cell damage is also a rationale for its use as a preconditioning treatment and as a hormetic agent, both of which are not included in routine clinical applications [21]. In a more recent study, it has been shown that upregulation of mRNA expressions and increases in activity of extracellular and Cu–Zn-SOD isoforms occur in long-term, intermittent exposures rather than acute, single-session HBO_2_ exposures [13]. Thus, the ability of endogenous antioxidant responses to prevent or ameliorate oxidative damage at significant levels is thought to be highly dependent on the pressure, duration, and frequency of HBO_2_ exposure, the general metabolic health of the tissue and organism, and individual antioxidant capacity [11,13,25]. Since there are no other factors that can affect the general metabolic health of the rats (diabetes mellitus, trauma, or infection) in the study design; it can be said that the decrease in SOD and GSH levels in the first 24 h and the return to the initial values at the end of the tenth day are mainly due to exposure pressure and frequency, in our study.

The significant erythrocyte GSH consumption in Group IV, however, is remarkable. GSH is a vital cellular redox buffer and it plays a pivotal role in the detoxification of not only MDA but also NO and other products of ROS-induced lipid peroxidation, such as 4-hydroxynonenal (4-HNE). Erythrocytes, which are continuously exposed to ROS in systemic circulation and autoxidation of hemoglobin in the cytosol, are important free radical scavengers. However, the plasma membranes of erythrocytes are very susceptible to oxidative stress damage due to very high unsaturated lipid percentages, which also provide considerable flexibility. Progressively increasing oxidative stress causes changes in hemoglobin structure and function, which may result in hemolysis. The replacement of depleted GSH cannot be performed by GSH itself since oral GSH is readily hydrolyzed by dipeptidases in the gastrointestinal tract and similarly, it is rapidly eliminated by gammaglutamyl transpeptidase in the circulation when administered as an infusion. Treatments targeting Nrf2 are therefore emphasized, one of which is HBO_2_. While only minimal changes were observed following treatment with NBO_2_, HBO_2_ has been reported to induce significant changes in Nrf2-induced antioxidant pathways [17,21,39,40]. On the other hand, it has been suggested that the induction of de novo GSH synthesis, subsequently the excessive ROS scavenging due to this enhanced activity following the cessation of hyperoxic exposure might also be responsible for “normobaric oxygen paradox” [23,41]. Furthermore, the interval between daily 30 min NBO_2_ exposures has been reported being extremely short to in the synthesis of sufficient erythropoietin to promote a significant increase in hemoglobin levels [41]. Compared to values at the end of the first day, the increase in erythrocyte GSH levels at the end of the tenth day of HBO_2_ in 2.4 ATA is significant in this respect.

## 5. Conclusions

In conclusion, the results of this study showed that three sessions of HBO_2_ exposures cause significant oxidative stress in the first 24 h, however, despite continued exposures on subsequent days, these effects are resolved at the end of the tenth day in the most preferred treatment protocol for acute HBO_2_ indications. In other words, HBO_2_-induced oxidative stress that does not kill healthy rats on the first day makes them stronger by triggering the signaling cascades and adaptive processes. In addition, exposure pressure and frequency are important factors to consider when investigating HBO_2_-induced oxidative stress and antioxidant response. There is a need for further studies to elucidate the molecular mechanisms of this biphasic response that HBO_2_ induces in acute cases and long-term.

## Figures and Tables

**Table 1:** Malondialdehyde (MDA), superoxide dismutase (SOD), and glutathione (GSH) levels (mean ± SD).

Groups (n = 8)	Plasma MDA (nmol/mL)	Plasma SOD (U/mL)	Erythrocyte GSH (mg/g Hb)
I	3.54 ± 0.39	36.31 ± 2.60	3.74 ± 0.19
II	4.15 ± 0.27	37.53 ± 2.26	3.69 ± 0.22
III	4.69 ± 0.38 *	28.55 ± 2.35 *	3.43 ± 0.32
IV	4.95 ± 0.44 *	27.18 ± 2.22 *	3.23 ± 0.24 *
V	3.71 ± 0.38	34.71 ± 2.35	3.71 ± 0.18
VI	3.65 ± 0.34	33.93 ± 3.33	3.71 ± 0.29
VII	3.31 ± 0.35	38.40 ± 4.26	4.09 ± 0.42

* *p* < 0.05, post hoc test with Dunnett comparisons against control group (Group I).

**Table 2:** Comparison of the Groups II with V, III with VI, and IV with VII.

Groups Compared *	MDA	SOD	GSH
II–V	0.036 **	0.040 **	0.674
III–VI	0.0009 **	0.006 **	0.115
IV–VII	0.0008 **	0.0008 **	0.002 **

* Compared by Wilcoxon Test, ** *p* < 0.05.
